# Peptide Vaccines as Therapeutic and Prophylactic Agents for Female-Specific Cancers: The Current Landscape

**DOI:** 10.3390/ph16071054

**Published:** 2023-07-24

**Authors:** Manju Lekshmy, Chandrasekharan Rajalekshmi Dhanya, Jayashree SatheeshKumar Smrithi, Janaki Anandavallyamma Sindhurani, Jiji Joseph Vandanamthadathil, Jayakrishnan Therthala Veettil, Leelamma Anila, Vishnu Sasidharan Lathakumari, Adhira M. Nayar, Maya Madhavan

**Affiliations:** 1 Department of Botany and Biotechnology, St. Xavier’s College, Thumba, Thiruvananthapuram 695586, Kerala, India; drlmanjuakathalam@gmail.com; 2 Department of Biochemistry, Government College Kariavattom, Thiruvananthapuram 695581, Kerala, India; dhanyasbabu@gmail.com; 3 TATA Translational Cancer Research Centre, Tata Medical Centre, Kolkata 700156, West Bengal, India; smrithi.js@ttcrc.tmckolkata.org (J.S.S.);; 4 Department of Biochemistry, NSS College, Nilamel, Kollam 691535, Kerala, India; sindhurani77@gmail.com; 5 Department of Zoology, Government Brennen College, Thalassery 670106, Kerala, India; jijijoseph@brennencollege.ac.in (J.J.V.); jkbrennen@brennencollege.ac.in (J.T.V.); 6 Department of Biochemistry and Industrial Microbiology, Sree Narayana College for Women, Kollam 691001, Kerala, India; vishnusncw@gmail.com; 7 Department of Zoology, Mahatma Gandhi College, Thiruvananthapuram 695004, Kerala, India; adhira.m.nayar@mgcollegetvm.org; 8 Department of Biochemistry, Government College for Women, Thiruvananthapuram 695014, Kerala, India

**Keywords:** female-specific cancers, peptide vaccine, HER2, non-HER2, HPV vaccine, prophylactic cancer vaccine, therapeutic cancer vaccine

## Abstract

Breast and gynecologic cancers are significant global threats to women’s health and those living with the disease require lifelong physical, financial, and social support from their families, healthcare providers, and society as a whole. Cancer vaccines offer a promising means of inducing long-lasting immune response against the disease. Among various types of cancer vaccines available, peptide vaccines offer an effective strategy to elicit specific anti-tumor immune responses. Peptide vaccines have been developed based on tumor associated antigens (TAAs) and tumor specific neoantigens which can also be of viral origin. Molecular alterations in HER2 and non-HER2 genes are established to be involved in the pathogenesis of female-specific cancers and hence were exploited for the development of peptide vaccines against these diseases, most of which are in the latter stages of clinical trials. However, prophylactic vaccines for viral induced cancers, especially those against Human Papillomavirus (HPV) infection are well established. This review discusses therapeutic and prophylactic approaches for various types of female-specific cancers such as breast cancer and gynecologic cancers with special emphasis on peptide vaccines. We also present a pipeline for the design and evaluation of a multiepitope peptide vaccine that can be active against female-specific cancers.

## 1. Introduction

For many years, the gold standard in cancer treatment is conventional methods like radiation therapy, chemotherapy, and surgery [1,2]. Consistent scientific effort has resulted in the development of a number of alternative potential treatment strategies to circumvent the therapeutic limitations of the current conventional methods [2,3,4,5]. Cancer cells are unique in their ability to bypass the immune system for their survival [6]. Activating the immune system to recognize and tackle tumors is a potentially effective therapeutic strategy against cancer. Several immunotherapeutic modalities for cancer include monoclonal antibody therapy [7,8,9] immune checkpoint blockade (ICB) [10], chimeric antigen receptor (CAR) T cell therapy [11], oncolytic viral therapy [12,13] natural killer (NK) cell therapy [14] and cancer vaccines [13,15]. Even though various immunotherapeutic approaches are available, cancer vaccines offer a promising method of inducing long-term immune response against cancer [16]. Among the various cancer vaccines available, peptide vaccines offer a promising strategy to elicit specific anti-tumor immune responses. This review focuses on immunotherapy, with special reference to peptide vaccines as therapeutic and prophylactic agents for treating cancers in women.

## 2. Female-Specific Cancers

The influence of cancer and its outcome on women needs special attention since it impacts the economic, emotional and social well-being of an individual extending to the society. Moreover, the enormous global discrepancies in female cancer survival make female-specific cancers a major public health concern [17]. A brief summary of the various aspects of female- specific cancers is depicted in Table 1.

## 3. Cancer Vaccines

Advances in bioengineering and material science have helped the development of different types of cancer vaccines which can arrest tumor progression and prevent recurrence [32]. An efficient cancer vaccine should ideally be able to reinforce the body’s natural defenses against cancer by eliciting potent CD4+ and CD8+ T effector and memory response [12]. Cancer vaccines come in a variety of forms which include cell-based vaccines/whole tumor cells [33,34], viral/bacterial-based vaccines [34,35,36], gene-based vaccines [13,34,37], and protein/peptide vaccines [34,38].

**Figure 1:**
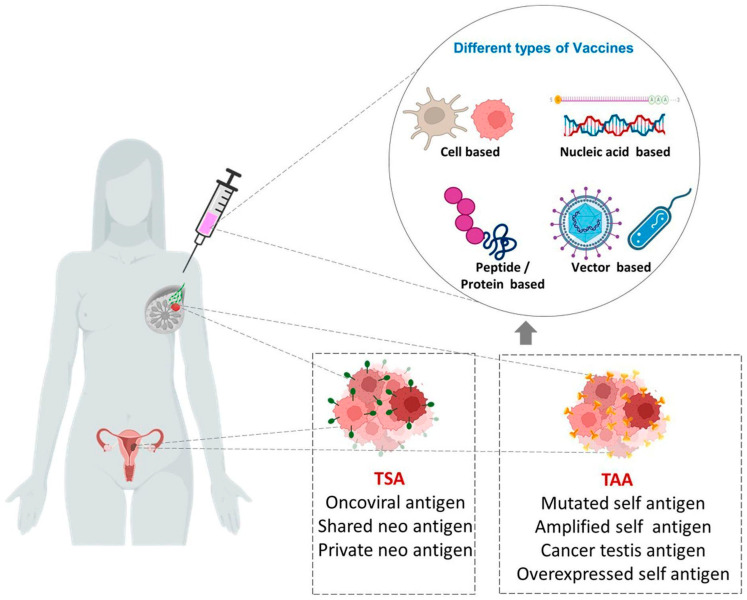
Different types of vaccines against female-specific cancers. TSAs (Tumor specific antigens) and TAAs (Tumor associated antigens) are exploited for the development of various types of vaccines such as Cell-based, Nucleic acid-based, Vector-based and Peptide/Protein-based vaccines.

Cancer vaccines stimulate both cellular and humoral immune response by utilizing tumor-associated antigens (TAAs) and tumor-specific antigens (TSAs), thus preventing tumor growth and killing tumor cells [39]. Traditional vaccine preparations that target TAAs are having low immunogenic capacity and risk of toxicity to normal cells [40]. TSAs or neoantigens, on the other hand, are only expressed by cancer cells and elicit strong immune responses because of the lack of immunological tolerance [41]. TSAs are highly specific and are used in the production and design of personalized vaccines [40]. The possibilities of various approaches in cancer vaccines with potential against female-specific cancers are summarized in Figure 1.

Vaccines based on peptides have an advantage over other forms of therapy. Metastatic cancers that have spread to various parts of the body can be treated with peptide vaccines, and they are nontoxic compared to other treatment strategies [42]. Unlike other immunotherapeutic techniques like CAR T cell therapy which targets a cell surface antigen, peptide vaccines are using multiple epitopes positioned outside or inside of tumor cells [43]. By developing a peptide vaccine devoid of B cell epitopes, the risk of hypersensitivity could be avoided, and highly heterogeneous tumors could be effectively targeted by peptide-based vaccines [44]. Though the role of B cell epitopes in cancer vaccine design is underappreciated, recent investigations reveal the significance of multiepitope vaccines encompassing B cell epitopes along with T helper and cytotoxic T lymphocyte (CTL) epitopes in prophylaxis and therapy of cancer [45].

## 4. Peptide Vaccines

**Figure 2:**
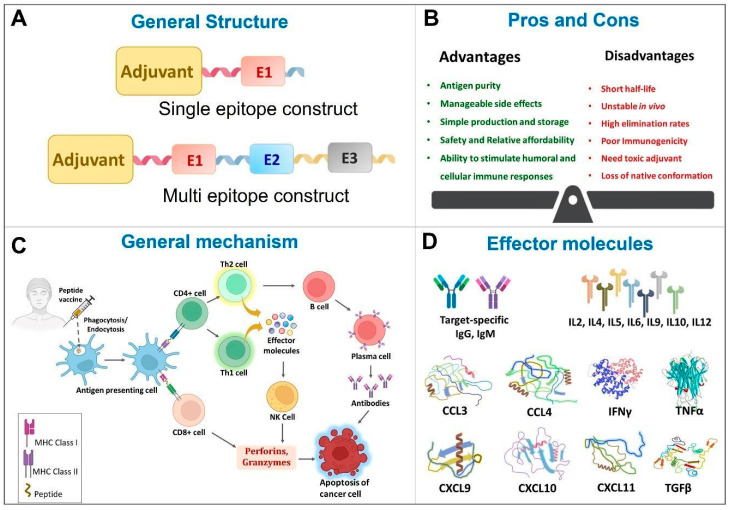
General features of Peptide vaccines. CCL—Chemokine (C-C motif) ligand; CXCL—Chemokine (C-X-C motif) ligand; E1—Epitope 1, E2—Epitope 2, E3—Epitope 3, IL—Interleukin; IFN—Interferon; NK—Natural killer; TGF—Tumor growth factor; Th—helper T cells; TNF—Tumor necrosis factor.

Peptide vaccines are composed of synthetic peptides which are highly immunogenic and elicit desired and specific adaptive immune response [46]. They come in a variety of forms, including multivalent long peptide vaccines, multi-peptide vaccines containing CTL and T helper-epitopes, peptide cocktail vaccines, hybrid peptide vaccines, personalized peptide vaccines, and peptide-pulsed dendritic cell vaccines [47]. The efficacy of peptide vaccines are widely studied against neurodegenerative diseases [48,49], infectious diseases [50] like human immunodeficiency virus (HIV) [51,52], hepatitis C virus [53], tuberculosis [54], foot and mouth disease [55], cancer [6], etc. The general aspects of peptide vaccines in the context of cancer therapy are summarized in Figure 2.

The differential expression of TAAs and TSAs on normal cells and cancer cells are made use of in designing peptide-based cancer vaccines [6,42,56]. Synthetic long peptides (SLPs) consist of 25–35 amino acids that are derived from TAAs or TSAs from a major type of peptide-based cancer vaccines [57,58]. Cancer vaccination trials with SLPs demonstrated inhibition of growth of transplanted tumors in mice [59]. Survivin-based vaccine, composed of a pool of three SLPs with eight CD4+ epitopes and six CD8+ epitopes, has shown to activate both CD4+ and CD8+ immune responses in mouse models for colorectal cancer. Fusion proteins made by combining Xcl1 with Ovalbumin SLP antigen and IgG1 Fc fragment were shown to elicit specific T cell response and sustained tumor control against the poorly immunogenic B16-OVA melanoma tumor [60].

Short peptides composed of 8–10 amino acids utilize Class I major histocompatibility complex (MHC) receptors and initiate CD8+ T cell response. Cancer neoepitope vaccine, based on MHC1 restricted short peptide, Nes2LR was reported to induce functional CD8+ T cell responses and prevent tumor growth in murine renal carcinoma model [61]

Recombinant overlapping peptides (ROPs) developed as a design strategy for peptide vaccines consist of a single-chain polypeptide with multiple epitopes. They can produce strong immunogenic responses in CD4+ and CD8+ T cells [62]. Immunoinformatics approaches were utilized to construct a multi-epitope peptide vaccine against breast cancer using immunogenic regions of the BORIS cancer-testis antigen containing multiple CTL epitopes. The selected regions were linked together by GPGPG linker followed by incorporation of T helper epitopes and the toll-like receptor (TLR)-4/MD-2 agonist. The resulting vaccine was reverse translated and then inserted into pcDNA3.1 to form the DNA vaccine [63]. Further investigations were carried out which revealed that co immunization of the multiepitope peptide vaccine and the resultant DNA vaccine significantly inhibited the growth of breast tumors, decreased tumor weight, inhibited metastasis, and enhanced survival time in murine mammary carcinoma [64].

## 5. Peptide Vaccines Developed for Female-Specific Cancers

### 5.1. Based on Genes Involved in Pathogenesis of Cancer

Accumulation of genetic and epigenetic alterations is well established to be involved in the process of carcinogenesis. The molecular alterations involved in pathogenesis of cancer include gene amplification, gene fusion, mutation and overexpression. Multiple studies have investigated the association of breast and gynecologic malignancies with overexpression and/or amplification of HER2 [65,66,67,68,69,70,71,72,73], as well as non-HER2 genes such as BRCA1/BRCA2, CHEK2, PTEN, MUCI, Tp53, MAGE, etc. [23,74,75,76,77,78,79,80]. The alterations identified in these genes are used successfully to design peptide vaccines with therapeutic efficiency against female-specific cancers.

#### 5.1.1. HER2 Based Peptide Vaccines for Female-Specific Cancers

HER2 is a receptor tyrosine kinase which is found to be involved in cell proliferation and survival [72]. Several HER2 derived peptide vaccines have been designed and are in the latter stages of clinical trials. B cell or T cell peptide-based vaccines, liposome-based vaccines with B cell peptides, and mature dendritic cells (DCs) loaded with TAA/TSA are a few of the diverse approaches employed [81].

HER2, a well-studied TAA is validated as a therapeutic target in breast cancer for the development of therapeutic vaccines. The present status of development of vaccines for breast cancer based on HER2 is summarized in Table 2.

Systematic review and meta-analysis were carried out to investigate the outcomes of HER2 based peptide vaccines in breast cancer. Both Chamani et al. [91] and You et al. [92] have reported that E75 vaccine is effective and safe in breast cancer while You et al. reported that GP2 vaccine elicited a strong immune response [92].

Overexpression, gene amplification and gene mutation of HER2 has been found to occur in patients with gynecologic malignancies with possible therapeutic implications [93]. In one of the earlier studies, intradermal immunization with a peptide vaccine based on HER2/neu combined with granulocyte macrophage colony stimulating factor (GM-CSF) as an adjuvant induced CD4+ T helper-specific immunological response in patients with breast and ovarian cancer. Patients produced HER2 specific T cell responses which could migrate out of the peripheral circulation [94].

Autologous DCs pulsed with HER2/neu or MUC1-derived peptides can effectively induce antigen-specific T cells in patients with advanced breast and ovarian cancer. The immunizations were shown to be well tolerated with no side effects in a pilot study involving 10 participants. MUC1 peptide specific T cells were found in patients vaccinated with HER2/new-derived peptides [95]. The uptake of tumor cells by DCs that are involved in cross-priming [96] and induction of other tumor antigen-specific CTLs could be the possible mechanism for this observation.

HER2/neu-specific antibody immunity was assessed in 35 patients with breast and ovarian cancer after immunization with HER2 based-peptides and successful immune response was recorded in majority of the patients. Moreover, epitope spreading to p53 was observed in 20% of the vaccinated patients [82]^.^

Monthly vaccination of 6 breast/ovarian cancer patients having HER2/neu-overexpressing tumors with HER2/neu-derived HLA-A2-peptide and GM-CSF as adjuvant, for six months was found to induce interferon-gamma (IFN-γ) secreting CD8+ T lymphocytes targeting HER2/neu. The minimal and transient nature of immune responses necessitated the need for CD4+ T cell support to maintain immunization [97].

In a Phase 1 study, 9 participants with epithelial ovarian, fallopian tube, or primary peritoneal carcinoma were administered with 5 class I MHC-restricted synthetic peptides derived from multiple ovarian cancer-associated proteins, as well as a class II MHC-restricted synthetic helper peptide derived from tetanus toxoid protein. All of the peptides used were immunogenic, including HER2/neu 754–762 peptide which stimulated CD8+ T cell responses. Authors suggested that the low potency of immunogenicity in ovarian cancers requires additional immunomodulation [98].

#### 5.1.2. Non-HER2 Based Peptide Vaccines for Female-Specific Cancers

Genes other than HER2 are also established to have a role in the pathogenesis of breast and gynecologic cancers. Mutations in MSH6, CHEK2, BRCA1, BRCA2, ATM, PMS2, PALB2, and MSH2 were found to occur more frequently than in any other gene in the analysis of breast and uterine cancer patients. The frequency of BRCA1, MLH1, MSH2, MSH6, PMS2, and PTEN mutations was higher in breast and uterine cancer than in breast cancer, whereas the frequency of ATM mutations was higher in breast and uterine cancer than in uterine cancer alone [99]. In 90% of mucinous ovarian carcinomas, KRAS, BRAF, and/or ERRB2 gene amplifications are present, demonstrating the therapeutic potency of RAS/MEK pathway in this subtype [100]. Table 3 describes the state-of-the-art progress in the development of peptide vaccines designed against non-HER2 genes for female-specific cancers.

### 5.2. Based on Viruses Involved in Pathogenesis of Female-Specific Cancers

Viruses are known to interact with host factors creating a tumor microenvironment (TME) that facilitates tumorigenesis [121]. Viruses are a possible cause of 15% of all human cancers, which is a sizable proportion of the worldwide cancer burden [122]. Human papilloma virus (HPV), Epstein-Barr virus (EBV), Mouse mammary tumor virus (MMTV) and Bovine leukemia virus (BLV) are known to be involved in the pathogenesis of female-specific cancers including breast and gynecologic cancers [123].

#### 5.2.1. Human Papilloma Virus (HPV)

HPV is a double-stranded, circular DNA [124] and the most prevalent sexually transmitted virus. High-risk HPV types (HPV16, 18, 31, 33, 35, 39, 45, 51, 52, 56, 58, 59) are the leading causes of genital tract cancers, including cervical, vulvar, vaginal, penile, and anal cancers, and a subset of head and neck cancers [125]. Subtypes 16, 18, and 33 are associated with 29% of breast cancer. [126] Two viral proteins, E6 and E7, are critical in initiating oncogenesis in infected cells, resulting in unregulated proliferation, unrestrained telomerase activity, and ultimately, cervical cancer progression [127]. HPV oncoproteins E6 can inactivate tumor suppressor protein p53 and E7 can inactivate pRb [128] which leads to the development of cancer.

The discovery of the etiologic involvement of HPV in the development and progression of cancers, mainly cervical cancer, has led to intensive research on prophylactic strategies. Currently available prophylactic vaccines exploit the ability of HPV capsid protein L1 to form virus-like particles (VLP) which are similar to native virions [129]. They induce the production of neutralizing antibodies that bind to viral particles and block their entry into host cells and effectively prevent HPV infections [130]. VLPs lack a viral genome and are neither infectious nor carcinogenic. In addition, they can provoke a robust humoral immune response with high and persistent neutralizing antibodies [131].

Three prophylactic peptide vaccines are commercially available against HPV infection, all of them based on L1 VLP. They are Gardasil^®^4, a quadrivalent vaccine [132], Cervarix™, a bivalent vaccine [133] and Gardasil^®^9, a nonavalent vaccine [133]. These prophylactic vaccines induce strong immune responses and produce high titer of antibodies [134]. HPV prophylactic vaccine is an ineffective treatment for an already infected person [135], but they can protect up to 100% of females between the ages of 9 and 26 from cervical cancer caused by HPV [136]. Table 4 enlists the currently available prophylactic peptide vaccines that could offer protection against HPV infection.

The clinical success of therapeutic cancer vaccines is very low due to the immunosuppressive nature of the TME. So, administration of cancer vaccine as a prophylactic measure in individuals at high risk of cancer or premalignant conditions will enhance the clinical efficacy [152].

Therapeutic vaccines for HPV are of four major categories such as live vector-based vaccines, peptide and protein-based vaccines, nucleic acid-based vaccines and whole cell vaccines [153]. peptide-based antigens are being extensively explored in the design of therapeutic vaccines against HPV infection.

In one of the initial studies, the impact of HPV16 E6 and E7 SLP vaccination on antigen-specific T cell response in cervical cancer patients was studied by Welters M J P et al. Patients were vaccinated with overlapping long peptides emulsified in Montanide ISA-51. Both CD4+ and CD8+ T cell responses to HPV16 E6 and E7 were observed [154].

In another study, women with HPV16 positive grade 3 vulvar intraepithelial neoplasia vaccinated with long peptides from the HPV16 viral oncoproteins E6 and E7 in incomplete Freund’s adjuvant showed strong interferon-γ–associated proliferative CD4+ T cell response and a broad response of CD8+ interferon-γ T cells. Positive outcomes appear to be linked to the activation of HPV16 specific immunity [155]. Studies indicated that mHSP110, a chaperone immunoadjuvant, enhanced the immune response to peptide vaccine based on HPV16 oncoprotein E7 derived CTL epitope E7 (49–57), inhibited tumor growth and prolonged survival time in mouse models for cervical cancer [156].

Cornelis et al. have reported that 20 women with high-grade vulvar intraepithelial neoplasia on receiving a synthetic peptide vaccine composed of 13 overlapping peptides with incomplete Freund adjuvant (mineral oil-based, Montanide ISA-51). In 9 individuals, the long-peptide vaccine completely regressed all lesions and eradicated HPV16. Clinical response was strongly linked with vaccine-induced T cell response [157,158].

A single administration of HPV vaccine having CpG oligodeoxynucleotides as an adjuvant and HPV16 E7 43–77 peptide as antigen was reported to elicit prophylactic and therapeutic effects on cervical cancer in mice models. Injection of vaccine increased cellular immunity mediated by CD4+ IFN-γ+ T cells and CD8+ IFN-γ+ T cells. Vaccine administration decreased numbers of immunosuppressive cells including regulatory T cells (Tregs) and myeloid-derived suppressor cells (MDSCs) [159]. Further studies were carried out with modified formulation of the vaccine in TC-1 grafted tumor. Subcutaneous injection of mannose-modified DCs-targeting liposomes loaded with HPV16 E7 peptide and CpG ODN vaccine stimulated powerful E7 specific CTL response and elevated the percentage of CD4+ T cells, CD8+ T cells and IFN-γ producing cells. Expression of IL-12, IFN-γ, TNF-α, and IL-2 were significantly increased, while those of IL-4 and TGF-β significantly decreased [160].

HPV16 E6/E7 synthetic overlapping long peptide vaccine was investigated for its therapeutic effect in high-grade cervical squamous intraepithelial lesions and was found to increase HPV16 specific T cell immunity which lasted up to one year [161] HPV16 SLP vaccination combined with Carboplatin and Paclitaxel chemotherapy was found to induce robust T cell response in a mouse tumor model and in patients with advanced cervical cancer [162]. Tri-therapy where HPV16 E7 SLP was administered, combined with Carboplatin/Paclitaxel followed by TLR9 agonist CpG resulted in regression of genital HPV16 tumors [163]. In 77 patients with advanced, recurrent, or metastatic cervical cancer undergoing Carboplatin/Paclitaxel chemotherapy, administration of ISA101, an SLP vaccine containing HPV E6/E7 showed type 1 T cell response and prolonged survival [164]. In a Phase I/II clinical study, immune responses of ISA101 vaccine with or without Polyethylene Glycol (PEG)ylated IFN-α as combination therapy with Carboplatin and Paclitaxel were evaluated. Enhanced tumor-specific immunity was observed and addition of PEGylated IFN-α enhanced the immune response [165].

A therapeutic vaccine for HPV called Pepcan (HPV16 E6 peptides combined with Candida skin testing reagent called Candin) was administered intradermally in 31 patients with high-grade squamous intraepithelial lesions and was reported to decrease the viral load in nine of the patients [166]. The Phase II clinical trial of Pepcan to the two treatment arms- Pepcan and Candin, in 99 patients with cervical high grade squamous intra-epitheial lesions was recently completed [167].

A bivalent therapeutic vaccine against HPV16/18 genotypes composed of a fusion protein containing the extra domain A of human fibronectin and HPV16/18 E7 viral antigens was developed by Arribillaga L et al. The vaccine induced E7-specific CTL response and eradicated pre-existing tumors [168]. Promising results have arrived from a Phase I clinical trial using the novel therapeutic vaccine, Hespecta (HPV E6 Peptide conjugated to Amplivant^®^) which showed a T cell specific immune response [169].

HPV16 E5 has also been proven to be a promising target for cervical cancer therapy. Administration of E5 peptide-based on epitopes predicted by immunoinformatics in combination with CpG has induced strong cell-mediated immunity, decreased tumor volume and increased survival time in mice models [170].

Administration of different immunodominant epitopes of HPV in combination was found to elicit increased immune response. Studies report induction of Th1 immune response and high Granzyme B secretion which indicates CTL activity in mice receiving E7 and E5 peptides together when compared to those receiving the peptides individually [171].

A multiepitope vaccine consisting of linked segments of E5, E6 and E7 peptides (E765m) was developed and inserted into the major immune dominant region (MIR) of hepatitis B virus core antigen (HBc) to form HBc-E765m chimeric virus-like particles (cVLPs). E5-TC-1 tumor-bearing mice immunized with cVLPs elicited high E5-, E6- and E7- specific CTLs, IgG antibody responses and increased levels of IFN-γ, IL-4 and IL-5. Tumor growth was also suppressed, which indicated that the novel vaccine provides a promising platform for immunotherapy in HPV16-associated cervical intraepithelial neoplasia [172]. An SLP vaccine containing HPV16 E7 antigen in combination with TLR9 agonist CpG formulated in an oil-in-water emulsion was found to inhibit tumor growth and induce robust CD8+ T cell response in TC-1 murine model [173].

Strategies using nanoparticles have been employed for the design of therapeutic peptide vaccines against HPV infection. Tat-E7/pGM-CSF nanoparticles are a promising new strategy for boosting the efficacy of peptide-based cervical cancer vaccinations. The HIV-1 Tat cell-penetrating peptide was fused with the HPV16 E7 CTL epitope and GM-CSF. In prophylactic and therapeutic mouse models, the vaccination resulted in lower tumor growth and improved long-term survival and higher frequency of CD8+ memory T cells [174]. In another study, Rahimian et al. used a double emulsion solvent evaporation technique to create polymeric nanoparticles (NPs) based on hydrophilic polyester loaded with an SLP derived from HPV16 E7 oncoprotein and a TLR3 ligand. There was a substantial increase in HPV specific CD8+ T cells when the HPV SLP antigen encapsulated in nanoparticles was administered. These biodegradable polymeric nanoparticles are an efficient alternative for adjuvant in cancer vaccinations since they cause no adverse reactions on administration [175].

A therapeutic HPV nanovaccine candidate was created by Zhang et al. using poly [d, l-lactic-co-glycolic acid] (PLGA), to encapsulate HPV-16 E744–62. Adenosine triphosphate (ATP) was added to the design as a novel adjuvant element. The PLGA encapsulation improved antigen presentation to antigen presenting cells (APCs), triggered the immunological response, ATP induced DC maturation, and improved antigen recognition and uptake by DCs [176]. Another novel liposomal nanoparticle based therapeutic peptide vaccine PDS0101 composed of cationic lipid R-DOTAP and 6 HPV16 E6/E7 peptides was developed. The ongoing clinical trial is evaluating the efficacy of this multipeptide vaccine when used in conjunction with chemotherapy and radiation therapy in 35 patients with stage IB3-IVA cervical cancer [177]. Future directions for HPV therapeutic vaccine development include the production of new potent adjuvants, novel antigen targets, and an enrichment of preclinical models.

#### 5.2.2. Mouse Mammary Tumor Virus (MMTV)

MMTV is a beta retrovirus that causes mammary cancers in both wild and laboratory mice [178] and has also been identified in 40% of human breast cancers [179]. It has been proposed earlier that the zoonotic transmission of MMTV from the mice, *Mus musculus domesticus* could account for the geographic differences in breast cancer incidence [180]. Stewart et al. has recently reported evidence for correlation of spikes in breast cancer incidence in Australia and New Zealand with mouse population outbreaks [181].

Ever since the involvement of MMTV with breast cancer was identified, investigations have been carried out to recognize TAA that can serve as potential vaccine targets. In one study using TgMMTV-neu mouse, three early-stage tumor antigens (PDHX, STK39, and OTUD6B) were identified by serological analysis of cDNA expression libraries (SEREX) screen that could serve as superior antigen targets for the inhibition of tumor growth [182]. MMTV-p14, the signal peptide of the MMTV envelope precursor, was found to be expressed on breast cancer cells. Protective vaccination using p14 with alum as an adjuvant revealed enhanced immune response which demonstrates p14 as a target for prophylactic vaccination in MMTV associated cancers [183]. Earlier, the feasibility of using MMTV-p14 for vaccination was demonstrated in Balb/c mice that harbor MMTV [184].

Proteins gp36 and gp52 which are part of the MMTV envelope were reported to be present in primary cultures of human breast cancer [185]. Protective efficacies of vaccines consisting of synthetic peptides based upon the primary sequence of gp52 were studied in mice models. Vaccinating Balb/c mice with surface accessible peptide region EP-3 of major viral envelope glycoprotein (gp52) of C3H-MuMTV was found to result in significant decrease in frequency of early onset tumors [186].

#### 5.2.3. Epstein-Barr Virus (EBV)

EBV is a DNA virus that belongs to the gamma Herpesviridae family [187]. The presence of the EBV genome was identified in a large subset of breast cancers by polymerase chain reaction (PCR), Southern blot analysis and immunohistochemical detection of Epstein-Barr nuclear antigen 2 (EBNA-2) [188]. Activation of HER2/HER3 signaling cascade is known to be involved in the malignant transformation induced by EBV [189].

Statistical association of EBV infection with increased breast carcinoma was demonstrated by meta-analysis [190]. Breast tumors showed viral products like EBNA-1, BZLF1, BARF-1, BARF-0, BXLF-2 and BFRF-3 [187]. Epidemiological studies suggest that EBV increases the risk for breast cancer and this association is stronger in Asian countries than in European countries, though EBV infection is not involved in the progression of breast cancer. Also, there is an association between EBV and breast cancer in areas where nasopharyngeal carcinoma is endemic [191].

Studies conducted by Li W et al. on the immune response of mice to EBV latent membrane protein 2 (LMP2) multi-epitope antigen demonstrated that priming with DNA vaccine and boosting with peptide vaccine elicited a robust humoral immune response and efficient CTL activity [192]. In vivo studies have reported that LMP-1 vaccines suppress LMP-1 expressing tumor growth and metastasis in nasopharyngeal carcinoma animal models [193]. Studies also suggest a correlation between expression of EBV LMP-1 and aggressive ER-negative breast cancer [194]. This opens a possible avenue for the development of LMP-1-based peptide vaccine as a therapeutic strategy against breast cancer.

The use of immunoinformatics approaches has resulted in the prediction of potential T cell and B cell epitopes for nine antigenic EBV proteins. The integrative meta-analytical approach could model these epitopes as effective candidates for peptide vaccine development towards the treatment of EBV associated cancers [195]. A computational meta-analysis integrated with dynamics could predict a panel of epitopes including B cell epitopes and cytotoxic T cell epitopes. These peptides were then docked against the MHC molecules and the selected peptides were subjected to molecular dynamics simulation and stability analysis. The validated peptides are suggested to aid in the development of vaccines that could be effective against multiple diseases caused by EBV [196]. A multiepitope based polyvalent vaccine against EBV associated tumors was developed using immunoinformatics approach. Molecular docking of the vaccine construct against TLRs revealed that it could elicit humoral and cellular immune responses [197].

#### 5.2.4. Bovine Leukemia Virus (BLV)

BLV is a delta retrovirus that most closely resembles human T cell lymphotropic virus 1 (HTLV-1) [198]. Association of breast cancer with exposure to BLV has been reported by Buehring G C et al. [199]. Literature search reveals a few investigations that have been carried out on development of peptide vaccines that could be effective against BLV associated tumors.

In one of the initial studies, Kabeya H et al. had reported that recombinant baculovirus (rgp51) and synthetic multiple antigenic peptides (MAP) of T helper, T cytotoxic, and B cell epitopes of BLV gp51 protected sheep from BLV [200].

Inoculation of mannan-coated liposome encapsulating 20-mer synthetic peptide of BLV envelope glycoprotein gp51 in BALB/c mice induced specific delayed-type hypersensitivity, lymphocyte proliferative responses, and weak cytotoxic lymphocyte response [201]. Glycoprotein gp51-peptide epitope covalently linked to a mutant bacteriophage carrier (mQβ) using two different linker strategies, isothiocyanate and dinitrophenyl adipate were reported to elicit long-lasting neutralizing antibodies in mice [202].

A prophylactic multi-epitope vaccine against BLV was computationally developed for breast cancer. The vaccine construct consisted of five antigenic CTL and four helper T lymphocyte (HTL) epitopes linked by AAY and GPGPG, respectively. β-defensin (TLR3 agonist) was added as an adjuvant using EAAAK linker. Immune simulation study confirmed that the designed vaccine could produce a higher response exhibited by helper T and cytotoxic T cell during vaccination. Also, NK and DCs demonstrated elevated macrophage activity [203].

An in silico approach was used to predict the reliable B and T helper cell epitopes of BLV that can be used for vaccine design. Immunogenic regions of linear and conformational epitopes were selected and the tertiary structure of the final epitope was modeled. The structures of both conformational epitopes were the same as that of the whole extracellular part of gp60 SU (surface glycoprotein of BLV, the major target for the host immunity against the virus) [204].

## 6. A Pipeline for the Design and Evaluation of Peptide Vaccine in Female-Specific Cancers

**Figure 3:**
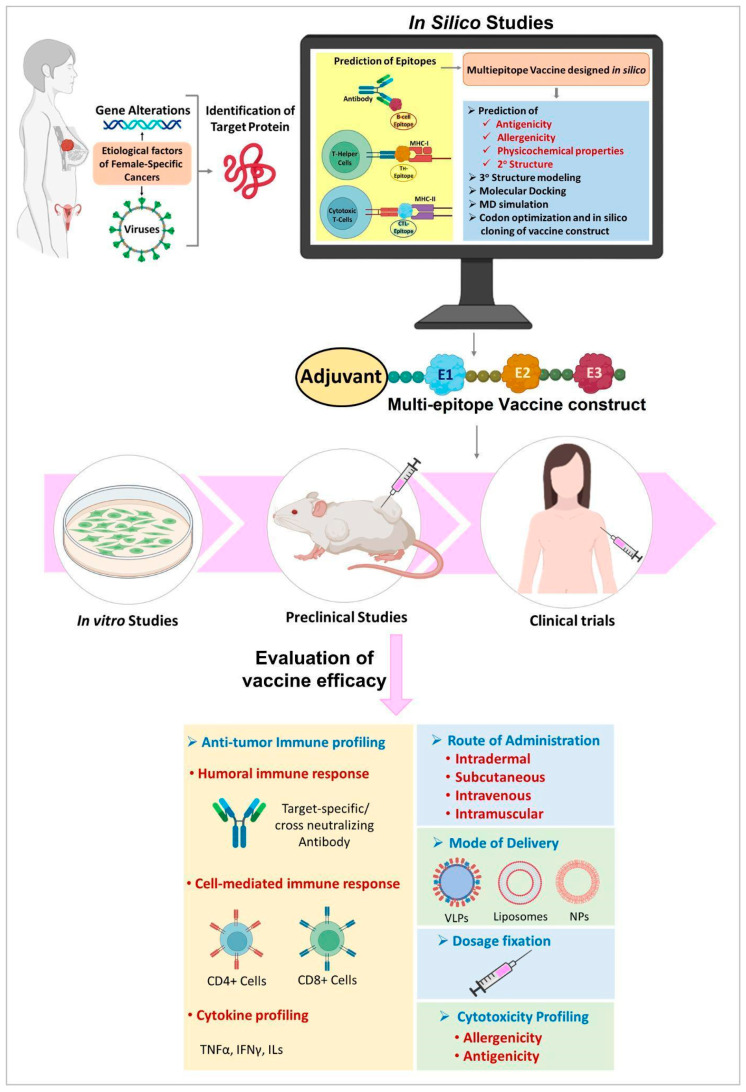
Study design for development of peptide vaccine. MD—Molecular dynamics, NP—Nanoparticle, VLP—Virus like particles.

One of the initial steps in the design of a peptide vaccine is the identification of appropriate epitopes with immunogenicity. Immunoinformatics approaches can be effectively used for the prediction of epitopes in vaccine research. The multi-epitope vaccine construct is an acceptable choice for future research [205]. Epitopes can also be designed based on the sequence of TAA or TSA that are encoded by mutated cancer genes [42,206,207]. In the case of viral induced cancers, the viral antigens can serve as a guide for epitope-based vaccine design [208,209,210]. After an epitope is predicted, a multi-epitope vaccine can be designed. Then the allergenicity, antigenicity and physicochemical properties of the vaccine construct is analyzed [211]. It is followed by preclinical or in vitro studies in cell lines and/or in animal models/humanized animal models and various phases of clinical trials before the successful development of a commercial vaccine. A flow chart illustrating a model pipeline for the design and evaluation of peptide vaccine in female-specific cancers is shown in Figure 3.

## 7. Delivery Systems for Peptide Vaccines

When used alone as vaccines, peptides do not elicit robust in vivo immune responses due to their rapid degradation at the injection site, lack of costimulatory effects and immune signals essential for APC activation [42]. In early phase clinical trials of peptide-based vaccines, overlapping long peptides were used along with adjuvants to boost immune response. For example, a vaccine made by combining HPV E6 and E7 peptides in Montanide ISA 51 adjuvant was well tolerated and induced the development of IFN-related T cell response in advanced cervical cancer patients [212]. So adjuvants and/or delivery systems are required to induce a satisfactory immune response and also protect the antigen from degradation and deliver it to the targeted cells. Delivery systems are self-adjuvating and they aid in the delivery of peptides to APCs to generate optimum T cell responses. Poly lactic-co-glycolic acid (PLGA) [213] and liposomes [214] are two drug delivery methods that have been studied for many years and have proven safety and efficacy for the treatment of cancer. Nanoparticles are an effective antigen presentation and delivery system for stimulating an optimal immune response [215]. The physical features of nanoparticles, such as size, shape, and surface characteristics can be easily modified to induce immunological responses against the associated antigen. Vaccines have been delivered via polymeric nanoparticles such as nanogels [216], dendrimers [216], hydrogels [217], and micelles [218] that have been conjugated with immune stimulants. Conjugation of peptides to inorganic nanoparticles like gold nanoparticles increases the stability and reproducibility of the conjugate [219]. A recent study by Firdaus et al. shows that a completely specified, natural, hydrophobic amino-acid-based polymer (Polyleucine) conjugated to peptide antigen works well for vaccine delivery mechanism [220]. VLPs can efficiently act as vehicles of antigens to APCs, where they are cross-presented in association with both MHC class I and class II molecules, eliciting both humoral and cellular immune responses [221]. Recently, adenovirus-inspired non-infectious VLP was shown to stimulate anti-tumor immune response in mouse modes of melanoma [222] present shortcomings of peptide vaccines necessitate additional research on more effective adjuvants, routes of administration, and novel delivery systems.

## 8. Limitations and Adverse Effects of Peptide Vaccines

Peptide vaccines provide a number of benefits, including simplicity of synthesis, low production costs, adaptability to antigens, and high specificity. However, they also have a number of drawbacks, including MHC constraints, poor immunogenic potency, and the necessity for an adjuvant. Even though stability and immunogenicity of peptide vaccines can be improved by conjugating them with adjuvants, the unwanted immune response elicited by the adjuvant is a challenge. The extent and diversity of MHC alleles in various populations and races also pose a barrier that a specific peptide may not induce much cell-mediated immunity in individuals with diverse MHC class I molecules. Since the vaccine must match the HLA in patients, a peptide vaccine for the entire human population cannot be designed due to the presence of HLA polymorphisms [223]. Even though peptides present on MHC-II molecules that are recognized by helper T cells could considerably improve efficacy, it is extremely difficult to predict the immunogenicity of MHC-II-restricted peptides due to their greater diversity and complexity than MHC-I-restricted peptides [224]. Short peptides may induce T cell tolerance since they can directly bind to MHC on non-professional APCs [225]. Also, the constrained conformation of short peptides will prevent them from folding into the three-dimensional structure that is required for proper immunogenicity [226,227]. Although algorithms for predicting T cell epitopes are widely employed, their accuracy and sensitivity are somewhat limited due to the fact that the spatial configuration of T cell epitopes changes when antigens bind to cell surface receptors. Consequently, false-positive and false-negative immune responses are possible [228]. Tumor-specific CD4+ T cell responses often target self-derived epitopes. This will hinder the immune system from realizing its entire potential in fighting against cancer, presenting another important challenge in peptide vaccine development [229].

## 9. Future Prospects

The increasing incidence of female-specific cancers across the globe remains a great challenge that needs to be addressed with therapeutic and prophylactic approaches. In the case of viral-induced female-specific cancers, the development of novel prophylactic vaccines is all the more important. Even though vaccinations against HPV are widely used, prophylactic strategies against retroviruses causing female-specific cancers, such as MMTV and BLV are not even in the nascent stages of development. The major concern regarding retroviruses is that they stably integrate into the host genome, enter long-term latency in some cells, and evade immune response making vaccination difficult [230].

Peptide-based vaccination approaches have several advantages over other forms of therapies in eliciting appropriate anti-tumor immune responses. This should be addressed in conjunction with the fact that the anticancer immune response of peptide vaccines can be attenuated by factors such as the complexity, continuous evolution of the TME and the influence of neoantigen-specific T cell immunity [231]. Also, due to their rapid degradation at the injection site, lack of costimulation, and lack of signals needed for APC activation, peptide vaccines may not induce robust immune reactions in vivo [232]. Thus more studies are warranted on the development of potent adjuvants or immunostimulators and efficient delivery systems that are capable of producing effective T cell responses.

The identification of optimal antigen targets, streamlining immunization regimens and exploring novel biomarkers that could predict the efficacy of vaccine response are all major domains that deserve additional attention in the near future [42]. Empirical research on the design and effectiveness of combining peptide-based cancer vaccination with other forms of existing therapy is also the need of the hour.

The concept of multi-epitope-based peptide vaccines is quite interesting for both therapeutic and prophylactic purposes. Advances in artificial intelligence should be effectively exploited for the development of new algorithms for the prediction of peptide-binding epitopes which would aid in the design of neoantigen-based cancer vaccines. Personalized peptide-based cancer vaccines, emerging as a promising strategy for eliciting a diversified antitumor immune response that is appropriate and useful to individual cancer patients, which is currently an expensive and time-consuming affair, also need more attention.

## Figures and Tables

**Table 1:** Various aspects of female-specific cancers.

Type of Cancer	Incidence Rate *** [18] (%)	Mortality Rate *** [18] (%)	Causative Factor/Agent		Treatment Strategy ** [19]	Post Treatment 5-Year Survival Rate. [20]		
			Pathogen	Gene Mutation		Localized Disease * (%)	Regional Spread * (%)	Distant Metastasis * (%)
Breast Cancer	58.5	17.7	Human Papilloma virus (HPV), Mouse Mammary Tumour Virus(MMTV) Epstein-Barr virus (EBV) [21]	BRCA1, BRCA2, TP53, PTEN, STK11, CDH1, PALB2, CHECK2, ATM, NBN, and NF1. [22,23]	Surgery, Radiation therapy, Endocrine therapy Chemotherapy, Targeted therapy, Immunotherapy.	99	86	30
Cervical Cancer	15.6	8.8	Sneathia, Pseudomonas, Ureaplasma urealyticum, Ureaplasma parvum, Chlamydia trachomatis, Trichomonas vaginalis, Atopobium HPV [24]	EGFR, KRAS and PIK3CA [25] CASP8, TMS1/ASC [26] ERBB3 [27]	Surgery, Chemotherapy, Targeted therapy, Immunotherapy.	92	59	17
Endometrial Cancer	10.8	2.5	Porphyromonas, Atopobium vaginae, Pelomonas, Prevotella. [24]	PIK3CA, PIK3R1, PTEN, KRAS, FGFR2, ARID1A (BAF250a), and CTNNB1 (β-catenin), MLH1, TP53 (p53), PPP2R1A, HER-2/ERBB2, PIK3CA, and PTEN [24]	Surgery, Chemotherapy, Targeted therapy, Hormone therapy, Radiation therapy	96	72	20
Ovarian Cancer	8.1	5.4	Proteobacteria/Firmicutes., HPV [24]	TP53, BARD1, CHEK2, RAD51, and PALB2, BRCA1 and BRCA2 [28]	Surgery, Chemotherapy, Targeted therapy, Immunotherapy	93	75	31
Vaginal cancer	0.46	0.21	Proteobacteria and Firmicutes, HPV [24]	TP53, KRAS, RASA1, KMT2D, and JAK2 [29]	Surgery, Chemotherapy, Radiation therapy.	69	57	26
Vulvar cancer	1.2	0.45	HPV [30]	PIK3CA, FBXW7, HRAS, FGFR3, STK11, AKT1, SMAD4, FLT3, JAK3, GNAQ, and PTEN [31]	Surgery, Chemotherapy, Radiation therapy, Targeted drug therapy, Immunotherapy.	86	53	19

*** GLOBOCAN 2020 crude rate in percentage (https://gco.iarc.fr/today/home (accessed on 7 April 2023)).** Treatment strategies as per www.mayoclinic.org (accessed on 7 April 2023) * Localized disease: The cancer is only in a specific part, without spreading to lymph nodes or nearby tissues. This includes stage I cancers, **Regional spread:** The cancer has spread to nearby lymph nodes or tissues, but has not spread to distant organs. This includes mainly stage II, III and IVA cancers, **Distant metastasis:** The cancer has spread to distant parts of the body such as the lungs, liver or bones. This includes stage IVB cancers. (https://www.cancer.org (accessed on 7 April 2023)).

**Table 2:** HER2 based peptide vaccines for breast cancer HER2/neu-derived GP2 and GP2–P4.

Peptide	Adjuvant	Mode of Administration	Therapeutic Strategy	Immune Response	Phase of Study	Phase of Clinical Trial	Study Sample	Reference
Three peptides of HER2/neu-(i) ECD; p42, p98 and p328 (ii) ICD p776, p927 and p1166 (iii) Peptides from both domains; p369, p688 and p971	GM-CSF	Intradermal	Mixed vaccine	HER-2/neu IgG specific antibody responses ↑	Clinical trial	Phase I	Stage III breast and ovarian cancer N = 38, Sub group: - breast cancer Stage III (n = 13) or IV(n = 18), ovarian (n = 5), or non small cell lung cancer (n = 2).	[82]
E75 peptide	GM-CSF	Intradermal	Monotherapy	Clonal expansion of E75-specific CD8+ T cells ↑ NPBC recurrence↓	Clinical trial	Two-Stage Safety Trial	Non-palpable breast cancer (NPBC) N = 53	[83]
GP2 peptide (HER2/neu, 654–662)	GM-CSF	Intradermal	Monotherapy	in vivo and ex vitro immune responses specific to GP2 ↑	Clinical Trial	Phase I	Disease free lymph node-negative, (HLA)-A2+ breast cancer N = 18	[84]
31 pooled peptide	Incomplete Freund’s adjuvant (Montanide ISA51)	Subcutaneous	In combination with chemotherapy, hormonal therapy and radiotherapy	CTL and/or IgG responses ↑	Clinical Trials	Phase II	Triple-negative breast cancer (TNBC) N = 79 Sub group: - mrTNBC group (n = 18), HER2-negative group (n = 41) and HER2-positive group (n = 18)	[85]
Triple peptide-MUC1 (159–167),CEA (605–613) and ErbB2 (368–377)	Montanide ISA 51	Subcutaneous	Multi peptide vaccine	IFN-γ producing CD8+ T cell response ↑	Clinical Trial	Phase I/II	High-risk disease-free ovarian and breast cancer N = 14 Sub group: - (ovarian n = 7 and breast n= 7).	[86]
9 MHC class I-restricted peptides from MAGE-A1, −A3, and -A10, CEA, NY-ESO-1, and HER2	TLR3 agonist, poly-ICLC and peptide derived from tetanus toxoid	Intramuscular and intradermal	Monotherapy	Peptide specific CD8+ T cell response ↑	Clinical Trial	Pilot study	Breast cancer patients with stage IB-IV resected. N = 12 Sub group: - (Estrogen receptor positive disease, n = 5 and HER2 amplified n = 5)	[87]
AE37 and GP2	GM-CSF	Intradermal	Monotherapy	AE37 specific CD4+ T cell response and GP2 specific CD8+ T cell response ↑	Clinical Trial	Phase II	Breast cancer with disease-free node positive and high-risk node negative patients N= 456	[88]
Mixed 19-peptide vaccine derived from 11 different TAAs including EGFR	Freund’s adjuvant (Montanide ISA-51VG; Seppic)	Subcutaneous	Monotherapy	Peptide specific Ig ↑	Clinical Trial	Phase II	Advanced metastatic triple-negative breast cancer (mTNBC) N = 14	[89]
HER2/neu-derived GP2 and GP2–P4	KLH	Subcutaneous	monotherapy	Humoral immune response— IFN-γ, IL-2, IL-4 and Th1 and Th2 ↑	Preclinical study	-	TUBO breast cancer model of BALB/c mice, overexpressing HER2/neu oncogene.	[45]
HER2/neu-derived peptide AE36	CpG-ODN	Subcutaneous	Monotherapy	Synthesis of cytokines ↑ CD8+ and CD4+ T cell responses ↑	Preclinical study	-	TUBO breast cancer model of BALB/c mice	[90]

AE37, AE36, E75 and GP2—immunogenic peptides from the HER2/neu; CpG-ODN—Oligodeoxynucleotides of cytosine and guanine; CTL—Cytotoxic T lymphocyte; ECD—Extracellular domain; EGFR—Epidermal growth factor receptor; GM-CSF—Granulocyte macrophage colony stimulating factor; ICD—Intracellular domain; IFN-γ—Interferon gamma; IgG—Immunoglobulin G; IL- Interleukins; KLH—keyhole limpet hemocyanin; MHC—Major histocompatibility complex; NPBC—Node-positive breast cancer; Poly-ICLC—Polyinosinic-polycytidylic acid; TAAs—Tumor-associated antigens; Th—T helper cells; TLR—Toll-like receptor; TNBC—Triple-negative breast cancer; TUBO—cells lines cloned from a BALB/c mouse mammary carcinoma. (‘N’ represents the size of the study sample and the ‘n’ represents the size of the subgroup within the main study group). ↑: increased; ↓: decreased.

**Table 3:** Non-HER2 based peptide vaccines for female-specific cancers.

Peptide	Adjuvant	Mode of Administration	Therapeutic Strategy	Immune Response	Phase of Study	Phase of Clinical Trial	Study Sample	Reference
HER-2/neu-derived E75/MUC1-derived M1.2 peptide	-	Subcutaneous	Autologous DCs pulsed with HER-2/neu– or MUC1-derived peptides	CEA- and MAGE-3 peptide-specific T-cell response ↑ MUC1-specific T lymphocytes ↑ MAGE-3- and CEA- peptide specific CD8+ T cell response ↑	Clinical trial	Phase I/II	Metastatic breast cancer expressing HLA-A2 and HER-2/neu or MUC1 N = 7 breast cancer and N = 3 ovarian cancers	[95]
MUC1 lipopeptide	Lipid A	Subcutaneous	Monotherapy	MUC1-specific CTL response↑ T cells expressing intracellular IFN-γ ↑ T cells reactive with H-2Db/MUC1 tetramer ↑	Preclinical	-	MUC1 Transgenic mice	[101]
p53- synthetic long peptide (SLP)	Montanide ISA51	Subcutaneous	Monotherapy	IFN-γ producing T-cells ↑ p53-specific Th1 and Th2 CD4+ T-cell responses ↑ Th1 and Th2 cytokines ↑ Circulating p53-specific T-cells ↓	Clinical trial	Phase II	Epithelial ovarian cancer N = 18	[102]
p53 SLP	Montanide ISA51	Subcutaneous	Immunization preceded by Cyclophosphamide administration	p53-specific T cells ↑ Th1 and Th2 cytokines ↑ p53-specific IFN-γ-producing T cells ↑	Clinical trial	Phase II	Epithelial ovarian cancer N = 19	[103]
Overlapping long peptides (OLP) from cancer-testis antigen NY-ESO-1	Montanide-ISA-51 in Poly-polyinosinic-polycytidylic acid (ICLC)	Subcutaneous	Monotherapy	NY-ESO-1–specific antibody ↑ NY-ESO-1–specific CD8+ T cells ↑ NY-ESO-1–specific CD4+ T cells ↑	Clinical trial	Phase I	Advanced ovarian cancer N = 11	[104]
p53-SLP^®^ vaccine	Montanide ISA51,	Subcutaneous	Vaccination followed by chemotherapy	p53-specific IFN-γ ↑	Clinical trial	Phase II	Epithelial ovarian cancer N = 17	[105]
p53 peptide	Montanide and GM-CSF	Subcutaneous Intravenous	Peptide admixed with Montanide and GM-CSF Peptide-pulsed dendritic cells	p53 specific immune response ↑	Clinical trial	Phase II	Ovarian cancer N = 13 (Subcutaneous administration) N = 6 (Intravenous administration)	[106]
Wilms’ tumor 1 (WT1) peptide	Montanide ISA 51	Intradermal	Monotherapy	WT1 peptide-specific delayed-type hypersensitivity (DTH) reaction ↑	Clinical trial	Phase II	Ovarian carcinoma N = 24 Cervical carcinoma N = 11 Uterine sarcoma N = 5	[107]
Cancer-testis (CT) peptide	-	Subcutaneous	Combination with 5 CT peptides	CT peptide- specific CTLs ↑	Clinical trial	Phase I	HLA-A24-positive patients with metastatic and advanced breast cancer. N = 9	[108]
Personalized peptide vaccine (PPV)	Montanide ISA51VG	Subcutaneous	PPV monotherapy/PPV in combination with chemotherapy	Peptide specific IgG responses ↑ Peptide specific CTL response ↑ Interleukin (IL)-6, C Reactive Protein (CRP) and Serum amyloid A (SAA) levels ↑	Clinical trial	Phase II	Recurrent ovarian cancer N = 42 Sub group: (Platinum-sensitive n = 17 and platinum resistant n = 25)	[109]
WT1 peptide/MUC1 long peptide/	OK-432,	Intradermal	Chemotherapy followed by DC-based immunotherapy	WT1-specific CTL ↑	Clinical trial	-	Recurrent ovarian cancer (ROC) N = 56 Sub-group: (Serous cystadeno carcinoma n = 37, Endometrioid adenocarcinoma n = 6, clear cell adenocarcinoma n = 5, other cancers n = 4	[110]
Five HLA-A2402-restricted epitope peptides from KOC1, TTK, URLC10, DEPDC1 and MPHOSPH1	Incomplete Freund’s.	Subcutaneous	Combination therapy with Cyclophosphamide	T cell response ↑ Overall survival ↑	Clinical trial	Phase I	Cervical cancer and other solid tumors N = 18 Subgroup: (cervical cancer n = 1, other solid tumors n = 17)	[111]
Folate receptor alpha	GM-CSF	intradermal	Combination therapy with low-dose Cyclophosphamide	T cell response ↑	Clinical trial	Phase 1	Stage II-III breast or stage II-IV ovarian cancer N = 22 Subgroup: (breast cancer n = 8 ovarian cancer n = 14)	[112]
Qβ-MUC1	Incomplete Freund’s adjuvant	Subcutaneous	Immunotherapy	IgG antibodies ↑	Preclinical study	-	MUC1.Tg mice	[113]
RNF43 peptide pulsed DCs	-	Subcutaneous	Combination therapy with low-dose Cyclophosphamide	Serum IL-6 level ↑ IFNγ-producing, tumor-reactive CD8+ T cells ↑ Treg Cells ↓	Clinical trial	Phase I	Cervical cancer and other solid tumors N = 10 Subgroup: (cervical cancer n = 1 other solid tumors n = 9).	[114]
Peptides derived from FOXM1, MELK, Holiday Junction Recognition protein VEGF receptors 1 and 2	Incomplete Freund’s adjuvant	-	Immunotherapy	FOXM1 and MELK specific T-cell responses ↑	Clinical trial	Phase I	Recurrent or persistent cervical cancer N = 21	[115]
DC vaccine pulsed with personalized peptides (PEP-DC) or with tumor lysate (OC-DC)	-	Intranodal	Carboplatin/Paclitaxel adjuvant chemotherapy; Immunomodulation with low dose Cyclophosphamide	Proposed epitope spreading, increase in pre-existing NeoAgs-specific T cell clones and immune response against patient-specific antigens	Clinical trial	Phase I/II	Advanced high-grade ovarian serous carcinoma N = 16	[116]
Folate-binding protein- derived E39 peptide	GM-CSF	Intradermal	Monotherapy	Disease free survival↑	Clinical trial	Phase I/IIa	Ovarian, endometria, fallopian, or peritoneal cancer N = 51	[117]
MUC1	TLR7 agonists	Intraperitoneal	Monotherapy	Cytokine production ↑ CD3+/CD8+ T-cells ↑ Antibody titre ↑ Tumor weight ↓	Preclinical study	-	4T1 mouse breast cancer cells, MCF-7 human breast cancer cells, MB231 human breast cancer cells BALB/c mice	[118]
MHC class I restricted neoantigen peptide-loaded DC vaccine	-	Intranodal	Immunotherapy	Neoantigen-specific T cell responses ↑ CA-125 levels ↓	Clinical trial	-	Chemorefractory ovarian cancer and malignant ascites N = 1	[119]
HSP90 derived MHC class II epitopes	Complete Freund’s adjuvant	Intravenous	Combined with STING agonist and/or anti CTLA-4 antibody	HSP90-specific IgG responses ↑ Th1 immune response ↑ CD8+ T cells response ↑ T reg cells ↑	Preclinical study	-	Breast cancer murine model	[120]

CA-125—Cancer antigen 125; CEA—carcinoembryonic antigen peptide; CRP—C-reactive protein; CTLA-4—cytotoxic T lymphocyte-associated antigen-4; CTLs—cytotoxic T lymphocytes; DC—Dendritic cell; DTH—Delayed type hypersensitivity; ER—Estrogen receptor; E75 and GP2—immunogenic peptides from the HER2/neu; GM-CSF—Granulocyte macrophage colony stimulating factor; HER-2—human epidermal growth factor receptor 2; HLA—Human leukocyte antigen; HSP—Heat shock protein; IFNγ—Interferon gamma; Ig—Immunoglobulin; IL—Interleukins; MAGE—Melanoma Antigen Gene; MUC1—Mucin short variant S1; OLP—Overlapping peptide; PEP-DC—DCs pulsed with up to ten peptides; RNF—Ring finger protein; ROC—recurrent ovarian cancer; SLP—synthetic long peptide; STING—stimulator of interferon genes; Th—T helper cells; TLR—Toll-like receptor; wt p53—Wild type p53; WT1—Wilms’ tumor 1. ↑: increased; ↓: decreased.

**Table 4:** HPV prophylactic peptide vaccines.

Commercial Name of Vaccine	Vaccine Composition			Effective against Which Type of Cancer	Acts against Which Strain	Dosage	Current Status of the Vaccine
	Peptides	Adjuvant	Other Components				
Gardasil	HPV—6L1, 11L1, 16L1, 18 L1 [137]	Aluminum (Amorphous Aluminum Hydroxyphosphate Sulfate), [137]	Sodium chloride, L-histidine, polysorbate 80, sodium borate [137]	Cervical, vulval, vaginal, and anal cancers and their associated precursor lesions (and a subset of head and neck cancers) Genital warts and laryngeal papillomas [138]	HPV types 6, 11, 16, 18 [139]	3 doses of 0.5-mL: intramuscularly at 0, 2 months, 6 months [139]	Licensed, Merck and Co. [137,140,141]
Cervarix	HPV—16 L1, 18 L1. [142]	AlSO4 (aluminium salt + MPL (3-O-desacyl-4′-monophosphoryl lipid A) [142]	Sodium chloride and sodium dihydrogen phosphate dihydrate [142]	Cervical, vulval, vaginal, and anal cancers and their associated precursor lesions (and a subset of head and neck cancers) [138]	HPV 16 and 18 [142]	3 doses of 0.5 mL, intramuscular injections at 0, 1, and 6 [138]	Licensed, GlaxoSmithKline [140,141]
Gardasil 9	HPV—6 L1, 11 L1, 16 L1, 18 L1, 31 L1, 33 L1, 45 L1, 52 L1, 58 L1 [143]	Aluminum (provided as AAHS), [143]	Sodium chloride, L-histidine, polysorbate 80, sodium borate [143]	Cervical, vulvar, vaginal, anal, oropharyngeal and Genital warts (condyloma acuminata) [143]	HPV-6, 11,16,18,31, 33,45,52, 58 [144]	2 or 3 doses Intramuscularly, depending on age at initiation [144]	Licensed Merck and Co. [140,141]
Cecolin	HPV—16 L1 18 L1 [145]	Aluminum hydroxide [145]	Phosphate buffered saline. [145]	Cervical cancer, CIN grade I-III and adenocarcinoma in situ (AIS). [145]	HPV 16/18 [140]	2-dose Intramuscularly for girls aged 9–14 years, 3-dose Intramuscularly for young women [146]	Licensed in China. WHO prequalification Status: Current [141,144,147]
EG-HPV	HPV—16 L1,18L1 [148]	CIA06 (Aluminum hydroxide + dLOS (CIA05) [148]	-	Cervical cancer [148,149]	HPV type 16 and type 18 [148]	3 doses at 0, 1, 6 [148]	Clinical phase I trial, Eyegene Inc. Korea [149]

AAHS—Amorphous aluminum hydroxyphosphate sulfate; CIN—cervical squamous intraepithelial neoplasia; dLOS (CIA06)—A novel proprietary immune adjuvant; EG-HPV—Combination of HPV 16 and 18 L1 VLP; HPV—Human papillomavirus; L1—Major capsid protein. Immunoinformatics and structural vaccinology approaches have led to the designing of a prophylactic HPV vaccine for protection against cervical cancer. The vaccine construct consisted of two immunodominant epitopes from L2 proteins of HPV, flagellin (TLR5 agonist), a short synthetic TLR4 agonist and T-helper agonists (PADRE and TpD) joined by appropriate linkers. The designed vaccine was suggested to elicit humoral and cellular immune responses and offer protection against HPV [150]. Efficacy of the vaccine was further established by in vivo experiments which demonstrated induction of IgG, Th1 (IFN-γ, IL-2) and Th2 (IL-4, IL-5, IL-10) type cytokines and elevated levels of IL-2 and IL-5 in vaccinated mice [151].

## Data Availability

Data is contained within the article.

## Nomenclature

AAHS	Amorphous aluminum hydroxyphosphate sulfate
AE37, AE36, E75 and GP2	Immunogenic peptides from the HER2/neu
APC	Antigen presenting cells
B16-OVA	B16 Ovalbumin
BLV	Bovine leukemia virus
BORIS	Brother of Regulator of Imprinted Sites
BRCA1/BRCA2	Breast Cancer gene
CA-125	Cancer antigen 125
CAR T cell	Chimeric antigen receptor T cell
CCL-Chemokine	(C-C motif) ligand
CEA	Carcinoembryonic antigen
CHEK2	Checkpoint kinase 2
CIN	Cervical squamous intraepithelial neoplasia
CpG-ODN	Oligodeoxynucleotides of cytosine and guanine
CRP	C-reactive protein
CTL	Cytotoxic T lymphocyte
CTLA-4	Cytotoxic T lymphocyte-associated antigen-4
cVLPs	Chimeric virus-like particles
CXCL	Chemokine (C-X-C motif) ligand
DCs	Dendritic cells
dLOS (CIA06)	A novel proprietary immune adjuvant
DTH	Delayed type hypersensitivity
E1, E2, E3	Epitope 1, 2, 3
EBNA-2	Epstein-Barr nuclear antigen 2
EBV	Epstein-Barr virus.
ECD	Extracellular domain
EGFR	Epidermal growth factor receptor
ER	Estrogen receptor;
ErbB2	Erythroblastic oncogene B
FR	Folate receptor
GM-CSF	Granulocyte-macrophage colony-stimulating factor
HBc	Hepatitis B virus core antigen
HER 2	Human epidermal growth factor receptor 2
HIV	Human immunodeficiency virus
HLA	Human leukocyte antigens
HPV	Human Papillomavirus
HSP	Heat shock protein;
HTL	Helper T lymphocyte
HTLV-1	Human T cell lymphotropic virus 1
ICB	Immune checkpoint blockade
ICD	Intracellular domain
IFN	Interferon
IgG	Immunoglobulin G
IL	Interleukin
KLH-	Keyhole limpet hemocyanin
LMP2	Latent membrane protein 2
MAGE	Melanoma Antigen Gene
MAP	Multiple antigenic peptides
MD	Molecular dynamics
MDSCs	Myeloid-derived suppressor cells
MHC	Major histocompatibility complex
MIR	Major immune dominant region
MMTV	Mouse Mammary Tumour Virus
MUC1	Mucin1
NK	Natural killer cell
NP	Nanoparticle
NPBC	Non-palpable breast cancer
NPS	Nelipepimut-S
OLP	Overlapping peptide
PCR	Polymerase chain reaction
PEG	Polyethylene Glycolylated
PEP-DC	DCs pulsed with up to ten peptides
Poly-ICLC	Polyinosinic-polycytidylic acid
PLGA	Poly lactic-co-glycolic acid
PTEN	Phosphatase and TENsin homolog
RNF	Ring finger protein; ROC-recurrent ovarian cancer
ROPs	Recombinant overlapping peptides
SEREX	Serological analysis of cDNA expression libraries
SLP	Synthetic long peptide
SSA	Serum amyloid A
STING	Stimulator of interferon genes
TAAs	Tumor associated antigens
TCR	T-cell receptor
TGF	Tumor growth factor
Th	helper T cells
TLR	Toll-like receptor
TNBC	Triple-negative breast cancer
TNF	Tumor necrosis factor
TME	Tumor microenvironment
Tp53	Tumor protein 53
TSAs	Tumor-specific antigens
TUBO	Cell lines cloned from a BALB/c mouse mammary carcinoma
VLP	Virus like particles.
wt p53	Wild type p53
WT1	Wilms’ tumor 1
